# The sensitivity and specificity of the toe brachial index in detecting peripheral arterial disease

**DOI:** 10.1186/1757-1146-6-S1-P3

**Published:** 2013-05-31

**Authors:** Peta Craike, Vivienne Chuter, Alan Bray, Ruth Keech, Richard Rownsley, Angela Carruthers

**Affiliations:** 1 School of Health Sciences, Faculty of Health, University of Newcastle, Australia; 2 Vascular Health Centre, Gateshead, Australia

## Background

Peripheral arterial disease (PAD) is reported to affect up to 12% of the adult population and has significant health ramifications. PAD is associated with delayed wound healing and amputation. Podiatric vascular assessment plays an important part of identifying PAD. Traditionally, ankle brachial indices (ABI) have been used as a screening tool for non-invasive assessment of PAD however recent evidence has suggested that in certain populations there may be a decrease in the ABI’s sensitivity and specificity. The toe brachial index (TBI) has been suggested as potentially a more reliable indicator of the presence of PAD however, there is limited evidence currently on the validity of the TBI.

## Methods

Participants were recruited from a Private Vascular Clinic, and a Community Health Centre Podiatry Service. ABI and TBI’s were performed on all participants. Colour duplex ultrasound (CDU) was used to determine the presence or absence of PAD. Diagnostic accuracy of ABI and TBI results were then determined through comparison with CDU scans.

## Results

56 participants were recruited to this study (M:40 F:16). The results of this study demonstrated that sensitivity for the presence of PAD was reduced in the ABI compared to the TBI measurements (47% and 95% respectively). The ABI measurement had higher specificity for detecting PAD than the TBI measurement (79% and 91% respectively).

## Conclusion

The results of this study indicate that the TBI measurement is more likely to detect the presence of significant PAD as diagnosed by CDU across a mixed population.

